# Feedback Power Control Strategies in Wireless Sensor Networks with Joint Channel Decoding

**DOI:** 10.3390/s91108776

**Published:** 2009-11-03

**Authors:** Andrea Abrardo, Gianluigi Ferrari, Marco Martalò, Fabio Perna

**Affiliations:** 1 Department of Information Engineering, University of Siena, Italy; E-Mails: abrardo@dii.unisi.it; perna@unisi.it; 2 Wireless Ad hoc and Sensor Networks (WASN) Laboratory, Department of Information Engineering, University of Parma, Italy; E-Mail: martalo@tlc.unipr.it

**Keywords:** sensor networks, source correlation, feedback power control, joint channel decoding (JCD), serially concatenated convolutional codes (SCCCs), low-density parity-check (LDPC) codes, multiple access interference, noisy feedback, central estimating officer (CEO) problem, fusion

## Abstract

In this paper, we derive feedback power control strategies for block-faded multiple access schemes with correlated sources and joint channel decoding (JCD). In particular, upon the derivation of the feasible signal-to-noise ratio (SNR) region for the considered multiple access schemes, i.e., the multidimensional SNR region where error-free communications are, in principle, possible, two feedback power control strategies are proposed: (i) a classical feedback power control strategy, which aims at equalizing all link SNRs at the access point (AP), and (ii) an innovative optimized feedback power control strategy, which tries to make the network operational point fall in the feasible SNR region at the lowest overall transmit energy consumption. These strategies will be referred to as “balanced SNR” and “unbalanced SNR,” respectively. While they require, in principle, an unlimited power control range at the sources, we also propose practical versions with a limited power control range. We preliminary consider a scenario with orthogonal links and ideal feedback. Then, we analyze the robustness of the proposed power control strategies to possible non-idealities, in terms of residual multiple access interference and noisy feedback channels. Finally, we successfully apply the proposed feedback power control strategies to a limiting case of the class of considered multiple access schemes, namely a central estimating officer (CEO) scenario, where the sensors observe noisy versions of a common binary information sequence and the AP's goal is to estimate this sequence by properly fusing the soft-output information output by the JCD algorithm.

## 1. Introduction

Wireless multiple access schemes, where correlated signals, observed at different nodes, need to be transferred to one or more collectors, model several communication scenarios. For example, these schemes apply to wireless sensor networks, where a set of nodes collect and transmit correlated data to a common sink [1]. In the case of a single collector node (the access point, AP), the design of efficient transmission mechanisms is often referred to as reach-back channel problem [2–4]. Assuming orthogonal additive white Gaussian noise (AWGN) channels between the nodes and the collector, the separation between source and channel coding is known to be optimal [4, 5]. This means that the theoretical limit can be achieved by, first, compressing each source up to its Slepian-Wolf (SW) limit and, then, utilizing independent capacity-achieving channel codes (one per source) [6]. In an attempt to exploit such correlation, many works have recently focused on the design of distributed source coding schemes that approach the SW fundamental limit on the achievable compression rate [7–10].

An alternative solution to distributed source coding is based on joint source channel coding (JSCC) schemes, where the correlated sources are not source encoded but only channel encoded. If one compares a JSCC system with a system based on source/channel coding separation with the same information rate, the channel codes used in a JSCC scheme must be less powerful (i.e., they have higher rates). In fact, this apparent weakness is compensated by exploiting the source correlation at the decoder, which jointly recovers the information signals of all sources. For this reason, this approach is also referred to as joint channel decoding (JCD). In this case, it can be shown that the final system performance can approach the theoretical limits. This approach has attracted the attention of several researchers in the recent past, also because of its implementation simplicity [11–15]. Note that, in the JCD approach, the sources are encoded independently of each other (i.e., for a given source neither the realizations from the other sources nor the correlation model are available at each encoder) and transmitted through the channel. In this case, the correlation between the sources has to be available at the (common) receiver.

In the introduced scenario, we study the performance of wireless multiple access schemes, with binary correlated sources communicating to an AP and with block faded communication links. It is well known that the presence of block-faded channels may dramatically degrade the performance of wireless multiple access systems, unless some countermeasures are taken at the transmitters to protect highly faded links. For instance, the performance of multiple access schemes can be improved by the use of “feedback.” In general terms, the AP can provide the sources with supplementary information (e.g., on the links' states) to allow them to counter-act the effects of fading. From an information-theoretic viewpoint, while feedback does not increase the capacity of a memoryless channel with one sender and one receiver [16], the capacity region of multiple access channels increases through the use of feedback [17, 18]. In [19, 20], the authors devise JSCC strategies for multiple-access channels with feedback and correlated sources.

In this paper, we refer to block faded multiple access schemes with correlated sources and JCD. In particular, we consider serially concatenated convolutional coding (SCCCing) or low-density parity-check (LDPC) coding at the sources. We first investigate, in the absence of non idealities (besides fading), feedback power control strategies which can guarantee theoretically error-free communications, i.e., that the system operational point lies in the feasible signal-to-noise ratio (SNR) region of the multiple access channel. In this context, we first propose a classical feedback power control strategy, which tends to balance (i.e., equalize) the link SNRs and is optimal in traditional transmitting scenarios where the sources are independent. Then, we derive an innovative optimized power control strategy, which makes the system operate in the feasible SNR region at the lowest transmit energy consumption. It will be shown that the latter strategy leads to “unbalanced” target SNRs at the correlated sensors and, to the best of our knowledge, this is a novel result. The impact of possible non-idealities, in terms of residual multiple access interference and noisy feedback channels, on the performance of multiple access schemes using the proposed feedback power control strategies is also investigated. Even in this case, it will be shown that the unbalanced SNR strategy is still to be preferred. Finally, we apply the proposed feedback power control strategies to a so-called central estimating officer (CEO) problem, which can be interpreted as a limiting case of the general class of considered multiple access scenarios [21]. In the considered CEO setting, the information sequences at the input of the sensor nodes correspond to noisy observations of the sequence output by a single binary source, and the AP's goal is to estimate the latter sequence. In this scenario, we derive a proper fusion rule to be applied, at the AP, after feedback power control. In particular, this does not entail any modification of the proposed feedback power control strategies, which can be directly utilized.

This paper is structured as follows. In Section 2., we describe the considered multiple access scheme. In Section 3., we first derive the power control strategies with unlimited transmit power, using both balanced SNR and unbalanced SNR strategies. Section 4. is devoted to the description of the JCD iterative decoding scheme at the AP and to the simulation-based performance analysis of the proposed multiple access schemes with feedback power control. In Section 5., we investigate the robustness of the proposed feedback power control strategies with respect to errors in the power control commands and the possible presence of residual multiple access interference. In Section 6., the proposed framework for multiple access schemes is extended to encompass, at the AP, the presence of information fusion after feedback power control, i.e., to a CEO scenario. Finally, in Section 7. we provide concluding remarks.

## 2. System Model in the Absence of Non-Idealities

### 2.1. Communication Scheme and Feedback Power Control

Consider *n* spatially distributed nodes which detect (i.e., receive at their inputs) binary information sequences ${\text{x}}^{\left(k\right)}=\left[x_{0}^{\left(k\right)},\ldots ,x_{L-1}^{\left(k\right)}\right]$, where *k* = 1, …, *n* and *L* is the signals' length (the same for all sources). The information signals are assumed to be temporally white with $P(x_{i}^{\left(k\right)}=0)=P(x_{i}^{\left(k\right)}=1)=0.5$ and the following simple additive correlation model is considered:

$$x_{i}^{\left(k\right)}=b_{i}⊕z_{i}^{\left(k\right)}\;i=0,\ldots ,L-1\;k=1,\ldots ,n$$

where {*b_i_*} are independent and identically distributed (i.i.d.) binary random variables and $\left\{z_{i}^{\left(k\right)}\right\}$ are i.i.d. binary random variables with probability *ρ* to be 0 (and 1 − *ρ* to be 1). Obviously, if *ρ* = 0.5 there is no correlation between the binary information signals ${\left\{{\text{x}}^{\left(k\right)}\right\}}_{k=1}^{n}$, whereas if *ρ* = 1 the information signals are identical. Note that in this paper, similarly to previous studies [11–15], we refer to a transmission scenario where quantization and digitization are performed separately. In this case, the original source correlation may be converted into correlation among the bit sequences of the binary digitized signals, as shown, for example, in [22]. Eventually, the correlation model between bit sequences is univocally determined by the probability that two corresponding (on a time scale) bits are equal. Therefore, we simply adopt this correlation model.

According to the chosen correlation model, the a-priori joint probability mass function (PMF) of the information signals at the inputs of the *n* sources at the *i*-th epoch (*i* ∈ {0, …, *L* − 1}), can be computed. After a few manipulations, one can show that [23]

(1) $$\begin{array}{ll} p({\text{x}}_{i}) & =p(x_{i}|b_{i}=0)p(b_{i}=0)+p({\text{x}}_{i}|b_{i}=1)p(b_{i}=1)\\ & =\frac{1}{2}\left[\rho^{n_{\text{b}}}{\left(1-\rho \right)}^{n-n_{\text{b}}}+{\left(1-\rho \right)}^{n_{\text{b}}}p^{n-n_{\text{b}}}\right]\;i=0,\ldots ,L-1 \end{array}$$

where ${\text{x}}_{i}=(x_{i}^{\left(1\right)},\ldots ,x_{i}^{\left(n\right)})$ and *n*_b_ = *n*_b_(***x****_i_*) is the number of zeros in ***x****_i_*. Under the considered correlation model, it is straightforward to express the joint entropy *H*(*n*) of the *n*-dimensional vector ***x****_i_* emitted by the *n* sources at the *i*-th epoch as follows:

(2) $$H(n)=-\frac{1}{2}\sum_{n_{\text{b}}=0}^{n}\left(\begin{array}{c} n\\ n_{\text{b}} \end{array}\right)\left[\rho^{n_{\text{b}}}{\left(1-\rho \right)}^{n-n_{\text{b}}}+{\left(1-\rho \right)}^{n_{\text{b}}}\rho^{n-n_{\text{b}}}\right]\cdot \log_{2}\left\{\frac{1}{2}\left[\rho^{n_{\text{b}}}{\left(1-\rho \right)}^{n-n_{\text{b}}}+{\left(1-\rho \right)}^{n_{\text{b}}}\rho^{n-n_{\text{b}}}\right]\right\}$$

**Figure 1.**
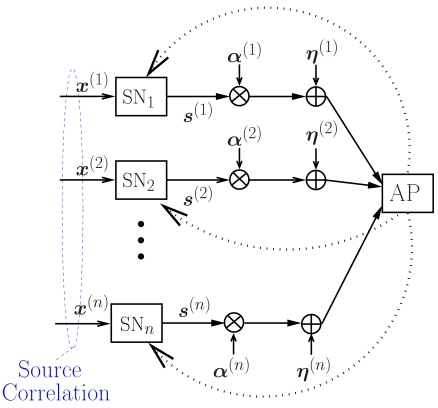
Multiple access scheme with feedback.

In Figure 1, the overall model for the multiple access scheme with feedback is shown. The goal of the communication system is that of recovering, at the AP, the information signals ${\left\{{\text{x}}^{\left(k\right)}\right\}}_{k=1}^{n}$ with the lowest possible probability of error. Referring to the equivalent low-pass signal representation, we denote as ***s***^(*k*)^ the complex samples transmitted by the *k*-th source and as *N* the length of ***s***^(*k*)^. In the remainder of this work, we will assume that the same transmitting rate *r* = *L*/*N* is used at all sources: however, the proposed approach is general and can be applied also to scenarios where the transmitting rate varies from source to source. By ${\text{α}}^{\left(k\right)}=[\alpha_{0}^{\left(k\right)},\ldots ,\alpha_{N-1}^{\left(k\right)}]$ we denote the complex gain vector over the *k*-th link, which encompasses both path loss and fading, and by ${\text{η}}^{\left(k\right)}=[\eta_{0}^{\left(k\right)},\ldots ,\eta_{N-1}^{\left(k\right)}]$ a complex AWGN vector. We assume a block fading model for the communication links between the sources and the AP: more precisely, the fading coefficient of each link is *constant* for the entire duration of a single packet transmission, i.e., $\alpha_{i}^{\left(k\right)}=\alpha^{\left(k\right)}$ for *i* = 0, …, *N* − 1. The fading coefficients are assumed to be independent from link to link and, on a single link, between consecutive packet transmissions (e.g., see note 1). Their amplitudes ${\left\{|\alpha^{\left(k\right)}|\right\}}_{k=1}^{n}$ are assumed to be Rayleigh distributed with 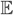
[|*α*^(*k*)^|^2^] = 1. We denote as ${\text{ν}}^{\left(k\right)}=[\nu_{0}^{\left(k\right)},\ldots ,\nu_{N-1}^{\left(k\right)}]$ the binary (not modulated) codeword $\left(\nu_{i}^{\left(k\right)}\in \{0,1\}\right)$ generated at the *k*-th node. For simplicity, we assume that binary phase shift keying (BPSK) is the modulation format, i.e., $s_{i}^{\left(k\right)}=y_{i}^{\left(k\right)}\sqrt{2E_{\text{c}}^{\left(k\right)}}$, where $y_{i}^{\left(k\right)}=2\nu_{i}^{\left(k\right)}-1=\pm 1$ and $E_{\text{c}}^{\left(k\right)}$ is the energy per coded bit transmitted by the *k*-th node. Indicating by $P_{\text{t}}^{\left(k\right)}$ the transmit power at the *k*-th node, the transmitted bit energy in the *k*-th link can be written as $E_{\text{c}}^{\left(k\right)}=P_{\text{t}}^{\left(k\right)}T_{\text{bit}}$, where *T*_bit_ is the bit duration. Since we are considering a block fading model, we assume that the link gains can be perfectly estimated at the AP (e.g., using a short preamble with pilot symbols).

We preliminary consider a system with orthogonal links. This is meaningful for wireless sensor networking scenarios with reservation-based medium access control (MAC) protocols, such as time/frequency division multiple access (TDMA/FDMA). The use of these protocols allows to represent the multiple access channel as a set of parallel orthogonal channels [4]. Therefore, in these cases the assumption of orthogonality is realistic. However, since in some cases orthogonality may be partially lost (e.g., in the case of non-orthogonal code division multiple access (CDMA) or in presence of FDMA with overlapping bandwidths), in Section 5. we will investigate the case where distributed users transmit simultaneously to the AP and, consequently, there appears multiple access interference at the AP.

Under the above assumptions, after matched filtering and carrier-phase estimation the real observable at the AP, relative to a transmitted sample, can be expressed as

(3) $$r_{i}^{\left(k\right)}=|\alpha^{\left(k\right)}|\sqrt{E_{\text{c}}^{\left(k\right)}}y_{i}^{\left(k\right)}+\eta_{i}^{\left(k\right)}\;i=0,\ldots ,N-1\;k=1,\ldots ,n$$

where $\eta_{i}^{\left(k\right)}$ is an AWGN variable with zero mean and variance *N*_0_/2.

Upon reception of the signals transmitted from all sources, the goal of the AP is to reconstruct each information signal by exploiting the source correlation. In order to do this, JCD schemes for two-source scenarios with systematic channel coding at each source and iterative decoding at the AP have been proposed [12, 14, 15]. In all cases, the key operational principle of the iterative decoder is that of using a soft-output component decoder per source and, then, to pass the generated soft-output values on the systematic bits (properly weighed taking into account the source correlation) to the other decoders. In Subsection 4.1., more details will be given and a general iterative decoder for an *n*-source scenario will be proposed.

In Figure 1, the feedback channels are indicated as dashed lines. In this work, we preliminary assume that these channels are error-free (i.e., each source perfectly receives the power control command sent to it by the AP) and then analyze the impact of noisy feedback (i.e., each source may receive a power control command different from that sent by the AP). More details on power control strategies with ideal and noisy feedback will be given in Section 3. and Section 5., respectively.

### 2.2. Feasible SNR Region of a Multiple Access Scheme

It is well known that distributed source coding allows to reduce the amount of data to be transmitted to the AP without needing extra inter-sensor communications. In particular, the performance achievable by distributed source coding is identical to that which could be achieved if the sources were encoded jointly. The SW theorem allows to determine the achievable rate region for the case of separate lossless encoding of correlated sources. Denoting by *r*_s,*k*_ the achievable compression rate for *k*-th transmitter, one obtains the following bounds:

(4) $$\sum_{m=1}^{p}r_{\text{s},k_{m}}\geq H\left(x_{i}^{\left(k_{1}\right)},x_{i}^{\left(k_{2}\right)},\ldots ,x_{i}^{\left(k_{p}\right)}|x_{i}^{\left(j_{1}\right)},x_{i}^{\left(j_{2}\right)},\ldots ,x_{i}^{\left(j_{n-p}\right)}\right)$$

where *p* ∈ {1, …, *n*}, *k_i_* ≠ *j_f_* (*i* ∈ {1, …, *p*}, *f* ∈ {1, …, *n* − *p*}), and {*k*_1_, …, *k_p_*}∪{*j*_1_, …, *j_n_*_−_*_p_*} = {1, …, *n*}. In other words, $H(x_{i}^{\left(k_{1}\right)},x_{i}^{\left(k_{2}\right)},\ldots ,x_{i}^{\left(k_{p}\right)}|x_{i}^{\left(j_{1}\right)},x_{i}^{\left(j_{2}\right)},\ldots ,x_{i}^{\left(j_{n-p}\right)})$ is the conditional joint entropy of the group of *p* sources indexed by *k*_1_, …, *k_p_*, conditioned on the remaining *n* − *p* sources.

By exploiting the well known relation between joint and conditional entropies [24], one gets:

(5) $$H\left(x_{i}^{\left(k_{1}\right)},x_{i}^{\left(k_{2}\right)},\ldots ,x_{i}^{\left(k_{p}\right)}|x_{i}^{\left(j_{1}\right)},x_{i}^{\left(j_{2}\right)},\ldots ,x_{i}^{\left(j_{n-p}\right)}\right)=H\left(x_{i}^{\left(1\right)},\ldots ,x_{i}^{\left(n\right)}\right)-H\left(x_{i}^{\left(j_{1}\right)},x_{i}^{\left(j_{2}\right)},\ldots ,x_{i}^{\left(j_{n-p}\right)}\right)$$

The considered correlation model between the sources is such that the joint entropy depends only on the number of considered sources, as shown in (2). Therefore, the family of inequalities in (4) can be equivalently rewritten as follows:

(6) $$\sum_{m=1}^{p}r_{s,k_{m}}\geq H(n)-H(n-p)$$

By assuming that source coding (compression) is followed by channel coding, the actual channel code rates ${\left\{r_{\text{c},k}\right\}}_{k=1}^{n}$ may be expressed as

(7) $$r_{\text{c},k}=r_{\text{s},k}\times r$$

where *r* is the (already introduced) transmission rate equal to *L*/*N*. The channel code rates must satisfy the following Shannon bounds:

(8) $$r_{\text{c},k}\leq \lambda_{k}\;k=1,\ldots ,n$$

where *λ_k_* is the capacity of the *k*-th link, i.e.,

(9) $$\lambda_{k}≜\frac{1}{2}\log_{2}(1+\gamma_{k})$$

and *γ_k_* is the SNR, at the AP, relative to the *k*-th link, i.e.,

(10) $$\gamma_{k}=\frac{{\left|\alpha^{\left(k\right)}\right|}^{2}E_{\text{c}}^{\left(k\right)}}{N_{0}}$$

As discussed in Section 1., compressing each source up to the SW limit and then utilizing independent capacity-achieving channel codes allows to reach the ultimate performance limits. Therefore, combining (6), (9), and (10), the link capacities ${\left\{\lambda_{k}\right\}}_{k=1}^{n}$ have to satisfy the following inequalities:

(11) $$\sum_{m=1}^{p}\lambda_{k_{m}}\geq r\left[H(n)-H(n-p)\right]\;p\in \{1,\ldots ,n\}\;\text{and}\;\{k_{1},\ldots ,k_{p}\}\subseteq \{1,\ldots ,n\}$$

From (11), using (9) it follows directly that the feasible *n*-dimensional SNR region of the considered multiple access scenario is characterized by the link SNRs at ${\left\{\gamma_{k}\right\}}_{k=1}^{n}$ such that, for any chosen value of *p* ∈ {1, …, *n*}, the following inequalities are satisfied:

(12) $$\sum_{m=1}^{p}\log_{2}(1+\gamma_{k_{m}})\geq 2r\times [H(n)-H(n-p)]\;\forall \{k_{1},\ldots ,k_{p}\}\subseteq \{1,\ldots ,n\}$$

**Figure 2.**
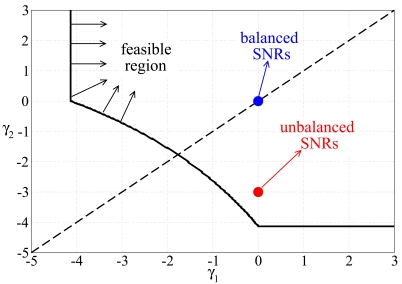
Feasible region in a scenario with two sources and *ρ* = 0.95.

In [25], it is shown that, if one solves (12) in a scenario with *n* = 2 sources and *ρ* = 0.95, the feasible SNR region shown in Figure 2 can be obtained. The target operational point of a feedback power control strategy can be represented as a point in this region. In Section 3., two feedback power control strategies for block faded scenarios will be proposed. The first one tries to make the system operational point lies on the bisector: in other words, *γ*_1_ = *γ*_2_ and, for this reason, this feedback power control strategy will be referred to as balanced SNR. The second selects the target SNRs so that the transmit energy consumption is minimized: in this case, it turns out that typically *γ*_1_ ≠ *γ*_2_ and, for this reason, this feedback power control strategy will be referred to as unbalanced SNR. In a general scenario with *n* sources, the balanced SNR feedback power control strategy will assume that all target SNRs are equal and properly select the common value, whereas the unbalanced SNR strategy will lead to different target SNRs over the links.

## 3. Feedback Power Control

### 3.1. Feedback Power Control Strategies with Unlimited Transmit Power

As discussed in Subsection 2.1., according to the considered block-faded sensor-AP channel model, the instantaneous per-link SNR at the AP is subject to per-packet fading fluctuations due to the time-varying nature of the channel. A feedback power control strategy for a multiple access scheme consists of a rule, depending on the (ideally perfectly estimated) links' statuses, according to which a power control command is sent, by the AP, to each sensor. Equivalently, the power control strategy is based on the determination of proper target SNRs at the AP, denoted as ${\left\{\gamma_{k}^{\left(\text{tgt}\right)}\right\}}_{k=1}^{n}$, for all sensors. On the basis of these target SNRs, the AP will send (without errors) the corresponding power control commands to the sources. Therefore, the *k*-th source will ideally set its transmit power in order to reach the target SNR at the AP. Under the assumption of unlimited transmit power at the sources, the transmit energy at the *k*-th node (assuming fixed bit duration) will be set as follows:

(13) $$E_{\text{c}}^{\left(k\right)}=\frac{N_{0}\gamma_{k}^{\left(\text{tgt}\right)}}{{\left|\alpha^{\left(k\right)}\right|}^{2}}=\frac{N_{0}\left(2^{2\lambda_{k}^{\left(\text{tgt}\right)}}-1\right)}{{\left|\alpha^{\left(k\right)}\right|}^{2}}$$

where $\lambda_{k}^{\left(\text{tgt}\right)}=\log_{2}(1+\gamma_{k}^{\left(\text{tgt}\right)})/2$ is the target capacity for *k*-th link. In this setting, a power control strategy consists in allocating the target SNRs ${\left\{\gamma_{k}^{\left(\text{tgt}\right)}\right\}}_{k=1}^{n}$ and, thus, the corresponding transmit energies ${\left\{E_{\text{c}}^{\left(k\right)}\right\}}_{k=1}^{n}$, so that the constraints (12) are satisfied for all users. Since the constraints (12) can be satisfied in infinite ways, we now propose two possible approaches: classical (in the sense of target SNR equalization over all links) and optimized (in the sense of overall transmit energy minimization).

The classical approach for power control in multiple access systems tries to “balance” the SNRs at the AP over all possible links. More precisely, the AP fixes a *common* target SNR, denoted as *γ̄*^(tgt)^, for all sources. Obviously, the common target SNR will have to be higher than the minimum required common SNR to guarantee that the operational point lies within the feasible SNR region introduced in Subsection 2.2.. The minimum common target SNR, denoted as *γ̄*^(tgt)−bal^, can be obtained by solving the following optimization problem with constraints given by (11):

(14) $$\begin{array}{lll} \text{minimize} & {\bar{\gamma }}^{\left(\text{tgt}\right)} & \\ \text{Subject to} & p\frac{\log_{2}(1+{\bar{\gamma }}^{\left(\text{tgt}\right)})}{2}\geq r\left[H(n)-H(n-p)\right] & p=1,\ldots ,n \end{array}$$

where we have used the fact that, under the considered common target SNR, it holds that

$$\lambda_{k_{m}}=\frac{\log_{2}(1+{\bar{\gamma }}^{\left(\text{tgt}\right)})}{2}\;\forall k_{m}\in \{1,\ldots ,n\}$$

From (14) one obtains, after a few mathematical passages, the following expression for the minimum common target SNR:

(15) $${\bar{\gamma }}^{(\text{tgt})-\text{bal}}=2^{\chi }-1$$

where

$$\chi ≜\underset{p=1,\ldots ,n}{\max}\left\{\frac{2r}{p}\left[H(n)-H(n-p)\right]\right\}$$

If multiple access schemes with uncorrelated sensors are considered, i.e., *ρ* = 0.5, it holds that *H*(*n*) = *n* and, therefore, *H*(*n*) − *H*(*n* − *p*) = *p*. In this case, the minimum target SNR in (15) reduces to

(16) $${\bar{\gamma }}^{(\text{tgt})-\text{bal}}=2^{2r}-1$$

In other words, the target SNR for each sensor is the minimum SNR which fulfills the Shannon capacity bound for a given rate *r*. Therefore, one can conclude that balancing the SNRs at the AP is optimal. On the other hand, it is straightforward to observe that for *ρ* > 0.5 it follows that *H*(*n*) − *H*(*n* − *p*) < *p*, i.e., *γ̄^(^*^tgt)−bal^ reduces with respect to the value for *ρ* = 0.5, thus allowing the system to reach a feasible point at lower energy consumption. However, in the presence of correlated sources, the above solution might no longer be optimal. This motivates one to investigate another power control strategy, as described in the following paragraph.

We now derive an optimized a transmit power allocation strategy which allows to achieve a feasible operational point at the lowest overall energy cost. In this case, the power control strategy can be cast into the following optimization problem with respect to the unknown vector of link capacities ***λ*** = (*λ*_1_, …, *λ_n_*):

(17) $$\begin{array}{lll} {\text{minimize}}_{\text{λ}} & f(\text{λ})=\sum_{k=1}^{n}\frac{\left(2^{2\lambda_{k}}-1\right)}{{\left|\alpha^{\left(k\right)}\right|}^{2}} & \\ \text{subject to} & \sum_{m=1}^{p}\lambda_{k_{m}}\geq r\left[H(n)-H(n-p)\right]\;p=1,\ldots ,n\;\{k_{1},\ldots ,k_{p}\}\subseteq \{1,\ldots ,n\} \end{array}$$

Once the solution ***λ***^(tgt)−unbal^ of the problem (17) is computed and recalling that $\lambda_{k}^{(\text{tgt})-\text{unbal}}=\log_{2}(1+\gamma_{k}^{(\text{tgt})-\text{unbal}})$, the transmit energy at the *k*-th sensor is allocated as follows:

(18) $$E_{\text{c}–\text{unbal}}^{\left(k\right)}≜\frac{N_{0}(2^{2\lambda_{k}^{(\text{tgt})–\text{unbal}}}-1)}{{\left|\alpha^{\left(k\right)}\right|}^{2}}=\frac{N_{0}\gamma_{k}^{(\text{tgt})–\text{unbal}}}{{\left|\alpha^{\left(k\right)}\right|}^{2}}$$

The problem (17) is a convex optimization problem which may be solved using standard convex optimization solvers [26]. It can be shown that in the case with *ρ* = 0.5 the optimal power control strategy derived in (17) returns the same target SNR shown in (16) for all users. Moreover, it is straightforward to observe that in the case of similar links, i.e., when the fading coefficients are the same in all links, the optimized power control strategy in (17) returns the same solution of the power control strategy (14). Hence, in this case as well the same target SNR is set for all sensors. In general, however, the optimized power control strategy leads to different target SNRs for the sources.

We now make a comment on the power control strategies described so far. The AP carries out its optimization strategy determining, after solving of (14) or (17), the target SNRs *at the receiver* (i.e., at the AP), which correspond to *γ̄*^(tgt)−bal^ or ${\left\{\gamma_{k}^{(\text{tgt})–\text{unbal}}\right\}}_{k=1}^{n}$, respectively. In the following sections, where the performance will be analyzed, we will consider the average SNR *at the transmitters*, which is defined as the arithmetic average of the actual SNRs (after power control) at the transmitters ${\left\{E_{\text{c}}^{\left(k\right)}/N_{0}\right\}}_{k=1}^{n}$, i.e.,

(19) $$\frac{{\bar{E}}_{\text{c}}}{N_{0}}≜\frac{\sum_{k=1}^{n}E_{\text{c}}^{\left(k\right)}}{nN_{0}}$$

Analyzing the performance as a function of the average SNR at the transmitters will be representative, for a given performance level, of the energy savings brought by each power control strategy.

**Figure 3.**
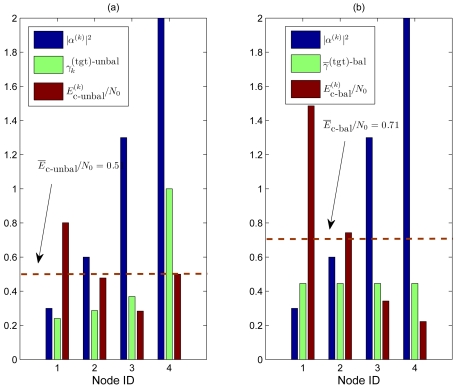
Illustrative comparison, in terms of target SNRs at the AP and at the transmitters, between (a) ideal unbalanced SNR and (b) ideal balanced SNR feedback power control strategies in a scenario with *n* = 4 sources and *ρ* = 0.95.

Before evaluating extensively, through simulations, the performance of the proposed feedback power control strategies in the following section, in Figure 3 we show an illustrative comparison, in terms of target SNRs at the AP and at the transmitters, between (a) (ideal) unbalanced SNR and (b) (ideal) balanced SNR feedback power control strategies in a scenario with *n* = 4 sources and *ρ* = 0.95. This is done in order to highlight the difference between the two strategies. In this illustrative example, the fading coefficients (depicted in blue) are distributed as follows: the fading coefficients of the first and second links are lower than 1 (|*α*^(1)^|^2^ = 0.3 and |*α*^(2)^|^2^ = 0.6, respectively), i.e., the fading affecting these links is strong, whereas the fading coefficients of the third and fourth links are higher than 1 (|*α*^(3)^|^2^ = 1.3 and |*α*^(4)^|^2^ = 2, respectively), i.e., the fading affecting these links is “beneficial.” At this point, we apply the two proposed power control strategies and we show the obtained target SNRs (at the AP) and the corresponding transmit SNRs at the sensors—in all cases, the SNRs are shown in a linear scale.

- In subfigure (a), we show the results obtained by applying the optimized (unbalanced) power control strategy. The target SNRs at the AP (shown as green bars) are the following: $\gamma_{1}^{(\text{tgt})-\text{unbal}}=0.24$, $\gamma_{2}^{(\text{tgt})-\text{unbal}}=0.29$, $\gamma_{3}^{(\text{tgt})-\text{unbal}}=0.37$, and $\gamma_{4}^{(\text{tgt})-\text{unbal}}=1$. At this point, the target SNRs at the transmitters (depicted in red) become
  $$\begin{array}{ll} \frac{E_{\text{c}-\text{unbal}}^{\left(1\right)}}{N_{0}}=\frac{{\bar{\gamma }}_{1}^{(\text{tgt})-\text{unbal}}}{{\left|\alpha^{\left(1\right)}\right|}^{2}}=0.8 & \frac{E_{\text{c}-\text{unbal}}^{\left(2\right)}}{N_{0}}=\frac{{\bar{\gamma }}_{2}^{(\text{tgt})-\text{unbal}}}{{\left|\alpha^{\left(2\right)}\right|}^{2}}=0.48\\ \frac{E_{\text{c}-\text{unbal}}^{\left(3\right)}}{N_{0}}=\frac{{\bar{\gamma }}_{3}^{(\text{tgt})-\text{unbal}}}{{\left|\alpha^{\left(3\right)}\right|}^{2}}=0.28 & \frac{E_{\text{c}-\text{unbal}}^{\left(4\right)}}{N_{0}}=\frac{{\bar{\gamma }}_{4}^{(\text{tgt})-\text{unbal}}}{{\left|\alpha^{\left(4\right)}\right|}^{2}}=0.5 \end{array}$$
  The average target SNR *Ē*_c−unbal_/*N*_0_ at the transmitter is thus equal to 0.51 (dashed horizontal line).
- In subfigure (b), we show the results obtained by applying the balanced SNR power control strategy. The minimum common target SNR at the AP, given by (15), is 0.45. Therefore, the target SNRs at the transmitters (depicted in red) are the following:
  $$\begin{array}{ll} \frac{E_{\text{c}-\text{bal}}^{\left(1\right)}}{N_{0}}=\frac{{\bar{\gamma }}^{(\text{tgt})-\text{bal}}}{{\left|\alpha^{\left(1\right)}\right|}^{2}}=1.5 & \frac{E_{\text{c}-\text{bal}}^{\left(2\right)}}{N_{0}}=\frac{{\bar{\gamma }}^{(\text{tgt})-\text{bal}}}{{\left|\alpha^{\left(2\right)}\right|}^{2}}=0.75\\ \frac{E_{\text{c}-\text{bal}}^{\left(3\right)}}{N_{0}}=\frac{{\bar{\gamma }}^{(\text{tgt})-\text{bal}}}{{\left|\alpha^{\left(3\right)}\right|}^{2}}=0.35 & \frac{E_{\text{c}-\text{bal}}^{\left(4\right)}}{N_{0}}=\frac{{\bar{\gamma }}^{(\text{tgt})-\text{bal}}}{{\left|\alpha^{\left(4\right)}\right|}^{2}}=0.23 \end{array}$$
  The average SNR *Ē*_c−bal_/*N*_0_ at the transmitter becomes 0.71 (dashed horizontal line).

The proposed illustrative comparison shows the benefits which can be obtained by properly unbalancing the target SNRs in the various links according to the actual channel conditions. In fact, in both power control strategies, setting the target SNRs at the transmitters as indicated makes the network operational point fall in the feasible SNR region, thus allowing theoretically error-free communications. The unbalanced SNR power control strategy, however, guarantees a given performance level at a lower energy cost than that required by the balanced SNR power control strategy.

### 3.2. Practical Feedback Power Control Strategies

The proposed feedback power control strategies require that a source might need to increase, in principle, its transmit energy without limit–for instance, this might be the case over a link characterized by an extremely small fading coefficient. Moreover, the proposed power control schemes assume the presence of an ideal communication (transmission and reception) scheme which achieves the system capacity bounds. Therefore, the power allocation strategies proposed so far will lead to reference performance results. In the remainder of this subsection, practical versions of the balanced SNR and unbalanced SNR feedback power control strategies are proposed, such that (i) the sources can adapt their transmit energies within a *limited* range ±Δ*E*_max_ at quantized steps of width Δ*E*_step_ and (ii) a proper energy gap, with respect to the ideal operational points, is considered.

For both practical versions of the proposed power control schemes, we assume that each node sends a pilot signal to the AP at a fixed initial SNR defined as follows:

(20) $$\frac{E_{\text{c}-\text{start}}}{N_{0}}≜\delta {\bar{\gamma }}^{(\text{tgt})-\text{bal}}$$

where *γ̄*^(tgt)−bal^ is the minimum target SNR given by the solution of the optimization problem (14) according to the balanced SNR power control strategy and *δ >* 1 is a coefficient which takes into account the non-idealities of the receiver (e.g., the suboptimal iterative decoding scheme). In other words, the starting target common SNR at the sensors would guarantee that the network operational point lies in the feasible SNR region in the absence of fading over the communication links. Note that *γ̄*^(tgt)−bal^ does not depend on the channel gains (as already observed) and, hence, may be assumed to be known at the transmitters—we are also implicitly assuming that the nodes know the variance of the AWGN at the AP. Upon receiving the pilot signals from all nodes, the AP is assumed to perform a perfect estimation of the fading coefficients and, therefore, of the received SNRs over all links:

$$\gamma_{k}=\delta {\bar{\gamma }}^{(\text{tgt})-\text{bal}}{\left|\alpha^{\left(k\right)}\right|}^{2}\;k=1,\ldots ,n$$

At this point, the AP compares, over each link, the received SNR with the corresponding target SNR, which depends on the chosen power control strategy. In the case of the balanced SNR power control strategy, the target SNR for the *k*-th source will be *δγ̄*^(tgt)−bal^ (i.e., the same for all sources); in the case of unbalanced SNR power control strategy, the target SNR will be $\delta \gamma_{k}^{(\text{tgt})-\text{unbal}}$. On the basis of the outcome of this comparison, the AP sends to the *k*-th source a power control command, in terms of required per-symbol energy variation Δ*E*_c,*k*_ (e.g., see note 2), according to the rule in Table 1, where the generic target SNR is denoted as $\gamma_{k}^{\left(\text{tgt}\right)}$ and depends on the chosen power control strategy, i.e.,

$$\gamma_{k}^{\left(\text{tgt}\right)}=\{\begin{array}{ll} \delta {\bar{\gamma }}^{(\text{tgt})-\text{bal}} & \text{balanced SNR}\\ \delta {\bar{\gamma }}_{k}^{(\text{tgt})-\text{unbal}} & \text{unbalanced SNR} \end{array}$$

In particular, we assume that the maximum per-bit energy variation which can be carried out by a source is Δ*E*_max_ (dimension: [dB]) and that the power control command is quantized with a step of Δ*E*_step_ (dimension: [dB]). The power control command transmitted back to the sources corresponds to a sequence of bits where each bit is “+1” for an “up” correction (+Δ*E*_step_) and “-1” for a “down” correction (−Δ*E*_step_). For instance, if the power control command is equal to +5 dB and Δ*E*_step_ = 1 dB, then the transmitted binary sequence is “+1+1+1+1+1.” The structure of the binary power control commands is shown in the last column of Table 1. In the following sections, the presented numerical results will be obtained considering Δ*E*_max_ = 20 dB and Δ*E*_step_ = 1 dB. Obviously, should Δ*E*_step_ → 0 and Δ*E*_max_ → ∞, the proposed practical power control strategies would reduce to the corresponding power control strategies with unlimited power control range. We assume that the end of the binary sequence operating the command is indicated by a proper end-of-command “flag.” We remark that, since we consider block faded channels, the power control command can be sent only once before each packet transmission.

It is worth noting that the proposed power control scheme compares favorably with classical power control schemes (see, for example, [27]), where the transmit power is continuously adjusted at fixed rate. By denoting as *T*_TPC_ the fixed transmit power control interval, classical power control schemes allow to compensate for a time varying fading provided that *T*_TPC_*f*_d_ ≪ 1, *f*_d_ being the fading Doppler frequency. Hence, the power control scheme proposed in this paper may be extended to time-varying channels provided that *T*_TPC_*f*_d_ ≪ 1 and the power control command is transmitted continuously during the packet. Obviously, in this case the overloading effect of feedback communications might be relevant and could affect the overall system performance. The extension of the proposed power control scheme to time-varying channels, and the corresponding impact of feedback overloading, is an interesting research direction and will be the subject of future work.

As for the reliability of the feedback power control command, in the presence of quasi-static channels it is reasonable to assume error-free feedback channels. More precisely, this can be obtained by protecting the power control commands through the use of low-rate channel codes, e.g., repetition codes. On the basis of these considerations, in Section 4. we will consider error-free (ideal) feedback channels. However, the impact of noisy feedback channels will be investigated in Subsection 5.2..

## 4. Performance Analysis in the Absence of Non-Idealities

### 4.1. Iterative Joint Channel Decoding at the AP

**Figure 4.**
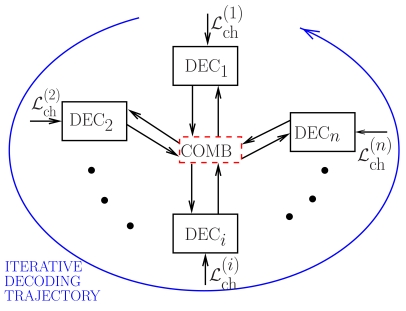
Iterative JCD scheme at the AP in the presence of *n* sources.

As described in Section 2., the information sequences are separately encoded using the same channel code (either an LDPC code or a SCCC) and transmitted over the communication links. In all cases, we assume that the common coding rate at the sources is *r* = *L/N* = 1/2. The proposed iterative decoding scheme at the AP is shown in Figure 4, where a channel decoder per source is considered and the trajectory of the iterative decoding process among the *n* component decoders is highlighted. The *i*-th component decoder, denoted as DEC*_i_* (*i* = 1, …, *n*), receives both the channel logarithmic likelihood ratios (LLRs) and the a priori probabilities obtained by properly processing the soft-output reliability values generated by the other component decoders. This processing/combining operation is carried out in the central block, denoted as “COMB,” where perfect knowledge of the source correlation (i.e., *ρ*) is assumed. At each component subdecoder, each coded sequence is decoded by using classical decoding algorithms; for instance, in the presence of an LDPC code the classical sum-product (SP) algorithm [28] is used, whereas in the presence of a SCCC turbo decoding, based on the use of the BCJR algorithm [29], is considered [30].

Under the assumption of perfect channel state information at the receiver, the channel LLR, relative to the *i*-th observable (*i* ∈ {1, …, *N*}) from the *k*-the node (*k* ∈ {1, …, *n*}), can be expressed as

(21) $${ℒ}_{i,\text{ch}}^{\left(k\right)}=\ln\frac{p(r_{i}^{\left(k\right)}|y_{i}^{\left(k\right)}=1,\alpha_{i}^{\left(k\right)})}{p(r_{i}^{\left(k\right)}|y_{i}^{\left(k\right)}=-1,\alpha_{i}^{\left(k\right)})}=\frac{2r_{i}^{\left(k\right)}\sqrt{E_{\text{c}}^{\left(k\right)}}\left|\alpha_{i}^{\left(k\right)}\right|}{\sigma^{2}}$$

where *σ*^2^ = *N*_0_/2. The maximum number of *internal* decoding iterations in each component decoder is denoted as $n_{\text{it}}^{\mathrm{int}-\max}$, and depends on the chosen channel coding (and decoding) scheme (e.g., see note 3). The a priori information about the correlation between the sources is exploited through an *external* iterative decoding process between the component subdecoders, and this process stops when a maximum number of external iterations (denoted as $n_{\text{it}}^{\text{ext}}$) is reached.

The total LLR relative to the *i*-th observable at the input of the *k*-th subdecoder can be expressed as follows:

$${ℒ}_{i,\text{in}}^{\left(k\right)}=\{\begin{array}{ll} {ℒ}_{i,\text{ch}}^{\left(k\right)}+{ℒ}_{i,\text{ap}}^{\left(k\right)} & i=0,\ldots ,L-1\\ {ℒ}_{i,\text{ch}}^{\left(k\right)} & i=L,\ldots ,N-1 \end{array}$$

In other words, the LLR of the *i*-th observable associated with an information bit (*i* = 0, …, *L* − 1) includes, besides the channel reliability value given by (21), the “suggestion” (represented by the soft reliability value ${ℒ}_{i,\text{ap}}^{\left(k\right)}$) obtained from a posteriori reliability values output by the other decoders. In particular, the a priori component of the LLR at the input of the *k*-th decoder can be written as

$${ℒ}_{i,\text{ap}}^{\left(k\right)}=\ln\frac{P(y_{i}^{\left(k\right)}=1)}{P(y_{i}^{\left(k\right)}=-1)}\;i=0,\ldots ,L-1$$

where $\left\{P(y_{i}^{\left(k\right)}=+1),P(y_{i}^{\left(k\right)}=-1)\right\}$ are derived from the soft-output values generated by the other decoders, as follows. In a straightforward manner, one can rewrite $P(y_{i}^{\left(k\right)})$ as

(22) $$P(y_{i}^{\left(k\right)})=\frac{1}{n-1}\underset{n-1\;\text{times}}{\underbrace{\left[P(y_{i}^{\left(k\right)})+\ldots +P(y_{i}^{\left(k\right)})\right]}}$$

Using Bayes' theorem [23], the probability $P(y_{i}^{\left(k\right)})$ can be expressed as

(23) $$P(y_{i}^{\left(k\right)})=\sum_{y_{i}^{\left(\ell \right)}=\pm 1}P(y_{i}^{\left(k\right)},y_{i}^{\left(\ell \right)})=\sum_{y_{i}^{\left(\ell \right)}=\pm 1}P(y_{i}^{\left(k\right)}|y_{i}^{\left(\ell \right)})P(y_{i}^{\left(\ell \right)})\;\ell =1,\ldots ,N\;\&\;\ell \neq k$$

Approximating the a priori probability $P(y_{i}^{\left(\ell \right)})$ in (23) with the *a posteriori* reliability value, denoted as $\hat{P}(y_{i}^{\left(\ell \right)})$, output by the *ℓ*-th decoder (*ℓ* ≠ *k*), from (23) one obtains:

$$P(y_{i}^{\left(k\right)})≃\sum_{y_{i}^{\left(\ell \right)}=\pm 1}P(y_{i}^{\left(k\right)}|y_{i}^{\left(\ell \right)})\hat{P}(y_{i}^{\left(\ell \right)})\;\ell =1,\ldots ,N\;\&\;\ell \neq k$$

where

$$\hat{P}(y_{i}^{\left(\ell \right)})=\{\begin{array}{ll} \frac{e^{{ℒ}_{i,\text{out}}^{\left(\ell \right)}}}{1+e^{{ℒ}_{i,\text{out}}^{\left(\ell \right)}}} & \text{if}\;y_{i}^{\left(\ell \right)}=+1\\ \frac{1}{1+e^{{ℒ}_{i,\text{out}}^{\left(\ell \right)}}} & \text{if}\;y_{i}^{\left(\ell \right)}=-1 \end{array}$$

where ${ℒ}_{i,\text{out}}^{\left(\ell \right)}$ is the soft-output a posteriori reliability on the *i*-th bit generated by the *ℓ*-th decoder. At this point, we evaluate the conditional probability $P(y_{i}^{\left(k\right)}|y_{i}^{\left(\ell \right)})$ in (23) d the *a priori* distribution (rather than a posteriori reliability values). By applying Bayes' theorem, it follows that

$$P(y_{i}^{\left(k\right)}|y_{i}^{\left(\ell \right)})=\frac{P(y_{i}^{\left(k\right)},y_{i}^{\left(\ell \right)})}{P(y_{i}^{\left(\ell \right)})}=2P(y_{i}^{\left(k\right)},y_{i}^{\left(\ell \right)})$$

where we have used the fact that $P(y_{i}^{\left(\ell \right)}=-1)=P(y_{i}^{\left(\ell \right)}=+1)=1/2$, since the BPSK symbols are supposed to be *a priori* equiprobable. Finally, the probability in (22) can be approximated as follows:

(24) $$\begin{array}{ll} P(y_{i}^{\left(k\right)}) & =\frac{1}{n-1}\left[\sum_{y_{i}^{\left(1\right)}=\pm 1}P(y_{i}^{\left(k\right)},y_{i}^{\left(1\right)})+\cdots +\sum_{y_{i}^{\left(k-1\right)}=\pm 1}P(y_{i}^{\left(k\right)},y_{i}^{\left(k-1\right)})+\sum_{y_{i}^{\left(k+1\right)}=\pm 1}P(y_{i}^{\left(k\right)},y_{i}^{\left(k+1\right)})+\cdots +\sum_{y_{i}^{\left(n\right)}=\pm 1}P(y_{i}^{\left(k\right)},y_{i}^{\left(n\right)})\right]\\ & ≃\frac{2}{n-1}\sum_{\begin{array}{l} \ell =1\\ \ell \neq 1 \end{array}}^{n}\sum_{y_{i}^{\left(\ell \right)}=\pm 1}\underset{\left[\text{from decoder}\;\ell \right]}{\underbrace{\hat{P}(y_{i}^{\left(\ell \right)})}}\cdot \underset{\left[\text{a priori source correl}.\right]}{\underbrace{\hat{P}(y_{i}^{\left(\ell \right)},y_{i}^{\left(k\right)})}} \end{array}$$

where $P(y_{i}^{\left(\ell \right)}|y_{i}^{\left(k\right)})$ can be obtained by marginalization of the *n*-th dimensional a-priori joint PMF $\left\{P(y_{i}^{\left(1\right)},y_{i}^{\left(2\right)},\ldots ,y_{i}^{\left(n\right)})\right\}$ of the information sequences at the input of the sources (e.g., see note 4). The intuition behind (24) consists in modifying the input a priori probability of a single bit by taking into account, through a weighed average, the reliability values (on the same bit) generated by the other decoders. In particular, the weight of the reliability value generated by the *ℓ*-th decoder is given by the joint a priori probability between the *k*-th and *ℓ*-th decoders.

It is immediate to observe that the proposed iterative JCD scheme has a complexity (measured in terms of basic operations, such as additions and multiplications) which, for a single external iteration, is a linear function of the number *n* of sources. In fact, there is a component decoder per source, so that there are *n* component decoders. The complexity required by each decoder, denoted as 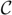
_dec_, depends on the specific decoding algorithm under use, namely the SP algorithm in the presence of LDPC coding or the BCJR algorithm in the presence of the SCCC. In both cases, the decoding complexity is linearly dependent on the number *N* of coded bits, i.e., one can write 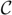
_dec_ = *N* 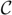
_dec−bit_, where 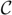
_dec−bit_ is the decoding complexity “per coded bit.” Finally, at the input of each decoder one needs to consider a proper combination of the LLRs on the information bits output by the other *n* − 1 decoders. This combination has a complexity which depends linearly on the number *L* of information bits per sequence. Indicating as 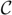
_LLR_ the complexity required to combine 2 LLRs, one can assume that the complexity required to combine *n* − 1 LLRs, relative to corresponding *n* − 1 bits at the same epoch, is on the order of *n* 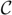
_LLR_. Therefore, recalling that the number of external iterations is $n_{\text{it}}^{\text{ext}}$, the overall complexity, denoted as 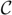, can be written as

$$\text{C}∼n_{\text{it}}^{\text{ext}}n(N{\text{C}}_{\text{dec}-\text{bit}}+nL{\text{C}}_{\text{LLR}})$$

where we use the symbol ∼ to loosely indicate “on the order of.” Since *L* = *Nr*, where *r* is the transmitting rate, the complexity can finally be expressed as follows:

$$\text{C}∼n_{\text{it}}^{\text{ext}}N(n{\text{C}}_{\text{dec}-\text{bit}}+n^{2}r{\text{C}}_{\text{LLR}})$$

As one can see, the complexity depends linearly on the number of external decoding iterations at the AP and on the sequence length, but it has a quadratic dependence on the number *n* of sources.

We remark that the above complexity level is realistic in the presence of digitization and decoding. In the case of real sources, one should perform more complicated (and computationally heavier) marginalization process to convert bit probabilities into symbol probabilities, and vice versa (see, for example, [22]). In this scenario, one could consider alternative schemes where real-valued phenomena are quantized and transmitted without resorting to channel coding/decoding and where detection and signal reconstruction are performed jointly on a single factor graph, as proposed in [31].

### 4.2. Numerical Results

In LDPC-coded scenarios, each of the source sequences is encoded using (i) a regular (3,6) LDPC code or (ii) an irregular LDPC code with double diagonal (DD) square submatrix in the parity check matrix [14]. The DD LDPC code provides a sort of implicit “differential encoding” effect which is in agreement with the design guidelines, presented in [32], for channel codes to be used in multiple access schemes. Both LDPC codes have a rate equal to 1/2 and *L* = 1000. Each component LDPC decoder at the AP performs a maximum number $n_{\text{it}}^{\mathrm{int}-\max}=50$ of internal iterations, whereas the fixed number $n_{\text{it}}^{\text{ext}}$ of external iterations between the decoders is set to 20. The LDPC codes are constructed in a *random* fashion, according to the following algorithm, which exploits an idea similar to the progressive edge growth (PEG) algorithm presented in [33]. Some potential connections, denoted as *sockets*, are drawn for all the variable and check nodes. Then, for each variable node a socket is randomly chosen, among all the free sockets at the check nodes, and the connection is added only if a *cycle* of a given (or lower) length is not created. In our case, the checked cycle length is equal to 6 for the regular LDPC code, whereas it is 4 for the DD LDPC code.

In turbo-like coded scenarios, we consider a SCCC given by the concatenation, through a bit random interleaver, of an outer convolutional code with an inner convolutional code [34]. The decoder is based on a message passing (turbo decoding) algorithm, such that the extrinsic information is iteratively passed between the inner and the outer soft-input soft-output (SISO) decoders for a predefined number *n*_it_ of iterations. The presence of a priori information coming from other decoders can be easily taken into account by feeding the a priori probabilities of information bits to the input of the outer SISO decoder in the form of LLRs. We consider the SCCC proposed in [25], which has been shown to perform very well in a scenario with JCD. More specifically, the SCCC is constituted by an outer 8-state rate-1/2 non-systematic and recursive convolutional code characterized by the following generator matrix:

$$G_{\text{outer}}(D)=\left[\frac{1+D^{2}+D^{3}}{1+D+D^{3}},\frac{1+D+D^{2}+D^{3}}{1+D+D^{3}}\right]$$

and by an inner 8-state rate-1/2 systematic and recursive code with the following generator matrix:

(25) $$G_{\text{inner}}(D)=\left[1,\frac{1+D+D^{2}+D^{3}}{1+D^{2}+D^{3}}\right]$$

The outer code is then punctured to obtain a rate equal to 3/4, while the inner code is punctured to obtain a rate equal to 2/3, so that the total rate of the SCCC is 1/2. The puncturing matrix *P*_o_ for the rate 3/4 outer code is

$$P_{\text{o}}=\left[\begin{array}{ccc} 1 & 1 & 0\\ 1 & 1 & 0 \end{array}\right]$$

which punctures (or erases) one third of the parity bits. The puncturing matrix *P*_i_ for the rate-2/3 inner code is

(26) $$P_{\text{i}}=\left[\begin{array}{cc} 1 & 1\\ 1 & 0 \end{array}\right]$$

which erases one half of the parity bits. In both outer and inner convolutional codes, the systematic bits are not punctured. In this case, each component decoder performs a fixed number $n_{\text{it}}^{\text{int}-\text{max}}=10$ of internal iterations, whereas the fixed number $n_{\text{it}}^{\text{ext}}$ of external iterations between the decoders is set to 5 (e.g., see note 5).

In all cases (LDPC-coded and SCCCed), we directly compare scenarios without feedback power control (W/o PC) and with feedback power control (W PC). Moreover, in the W PC case the performance is evaluated considering the two proposed power control strategies (balanced SNR and unbalanced SNR) and different values of the number *n* of sources (namely 2, 3, and 4). The maximum energy correction Δ*E*_max_ is set to 20 dB and *ρ* is set to 0.95. We have performed other simulations for different values of *ρ* and found similar results to those presented in this paper for values of *ρ* higher than 0.8. The value Δ*E*_max_ = 20 dB makes the improvement brought by the use of feedback power control strategies noticeable. Obviously, when Δ*E*_max_ decreases, this improvement reduces. The results presented in the following are obtained through computer simulations and collected by considering increasing values of the energy gap *δ*: for each value of *δ* we run a set of simulations for both balanced SNR and unbalanced SNR power control schemes. At the end of each simulation, we evaluate the average actual SNR at the sensors (after feedback power control), denoted as *Ē*_c_/*N*_0_, considering all *n* data flows and all fading realizations. Together with *Ē*_c_/*N*_0_, we also evaluate the average bit error rate (BER) and the outage probability (denoted as *P*_O_). As for the average SNR, the average BER is evaluated by averaging over all *n* data flows and all fading generations. Concerning the outage probability, an outage event occurs when at least one bit in at least one of the *n* packets from the sources to the AP is in error. The outage probability is thus evaluated by averaging the numbers of outage events over all fading generations.

**Figure 5.**
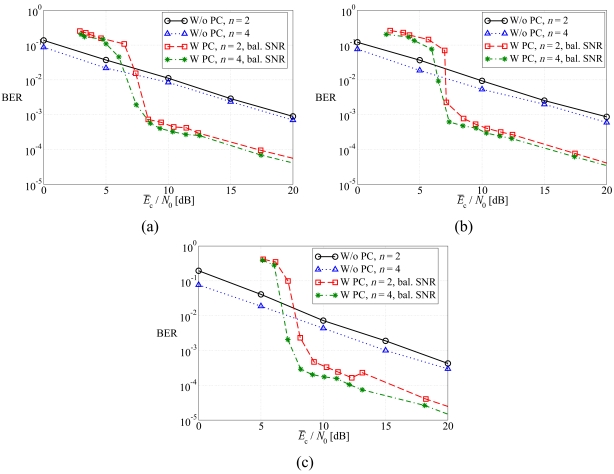
Average BER, as a function of the average SNR at the sources, considering: (a) regular LDPC-coded schemes, (b) DD LDPC-coded schemes, and (c) SCCCed schemes. The performance in the presence of the balanced SNR power control strategy is compared with that associated to the absence of power control.

In Figure 5, the average BER is shown, as a function of the average SNR at the sources, considering (a) regular LDPC-coded schemes, (b) DD LDPC-coded schemes, and (c) SCCCed schemes. The performance in the presence of the balanced SNR power control strategy is compared with that associated to the absence of power control. First, one can observe that, as expected, the use of feedback power control allows to significantly improve the performance with respect to scenarios without its use. As expected, when the number of sources increases, the BER reduces, since there exists a large number of communication links and, consequently, the high BER at the output of the strongly faded links can be partially lowered thanks to the reliable a-priori information coming from the soft-output decoders associated with the other sources which experience less faded links. When power control is considered, three different operating regions can be identified. For low values of the SNR, the performance of feedback power control schemes is worse than that without power control, since the transmit power is not sufficient to reduce the BER. Then, there exists an intermediate SNR region, with waterfall-like BER, where the system is able to (almost) completely compensate the faded communication links and, therefore, the performance is mainly limited by the error correction capabilities of the channel code. For large SNR, the slope of the BER curves decreases. In fact, in this operating region the performance is mainly limited by the fading fluctuations which do not allow to achieve the desired SNR, on account of the limited power control dynamics. Hence, the slope of the curves with power control is essentially the same of that with no power control and one can conclude that the unbalanced SNR power control strategy is not effective.

**Figure 6.**
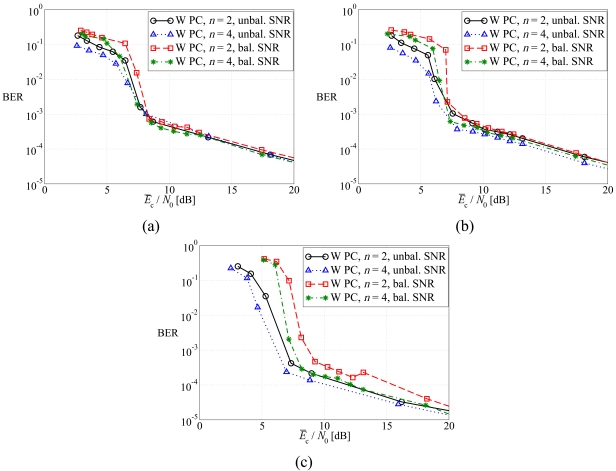
Average BER, as a function of the average SNR at the sources, considering: (a) regular LDPC-coded schemes, (b) DD LDPC-coded schemes, and (c) SCCCed schemes. Both balanced SNR and unbalanced SNR power control strategies are considered.

In Figure 6, the average BER is shown, as a function of the average SNR at the sources, considering (a) regular LDPC-coded schemes, (b) DD LDPC-coded schemes, and (c) SCCCed schemes. Both balanced SNR and unbalanced SNR power control strategies are considered. Motivated by the derivation in the previous sections, the unbalanced power control strategy is expected to guarantee, at a given SNR, a performance better than that with balanced SNR power control strategy. However, this effect can be slightly perceived when a regular LDPC code is used, whereas the gain becomes more evident when a DD LDPC code and, even more, a SCCC are used. In particular, the SCCC, in the presence of unbalanced SNR power control, allows to achieve a gain of more than 1 dB in the BER waterfall region with respect to the other two coding schemes. This significant impact of the designed channel code is in agreement with the information theoretic results presented in [25]. Indeed, the SCCC is shown to achieve a feasible SNR region larger than those of the LDPC codes and, therefore, it can exploit better the potential benefits brought by the use of the unbalanced SNR power control strategy, which is designed for an ideal scenario.

**Figure 7.**
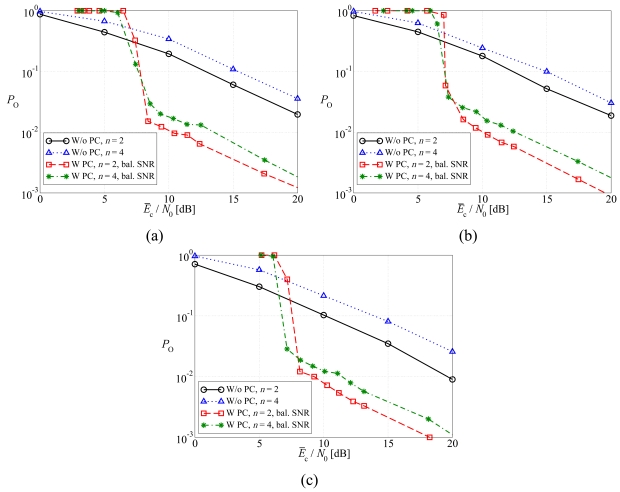
Outage probability, as a function of the average SNR at the sources, considering: (a) regular LDPC-coded schemes, (b) DD LDPC-coded schemes, and (c) SCCCed schemes. The performance in the presence of the balanced SNR power control strategy is compared with that associated with the absence of power control.

In Figure 7, the outage probability is shown, as a function of the average SNR at the sources, considering (a) regular LDPC-coded schemes, (b) DD LDPC-coded schemes, and (c) SCCCed schemes. The performance in the presence of the balanced SNR power control is compared with that associated to the absence of power control. Considerations similar to those carried out for the average BER still hold. In this case, however, a significant difference can be highlighted with respect to the performance in terms of average BER. In fact, the outage probability increases when the number of sources *n* increases (e.g., from 2 to 4). This is due to the fact that when the number of sources and, consequently, of transmitted packets increases, it is more likely that at least a bit is in error. On the other hand, if the outage probability is the performance metric of interest, the beneficial presence of a-priori information from the other (less faded) links is less noticeable than in the BER-based analysis, and the worst link dominates.

**Figure 8.**
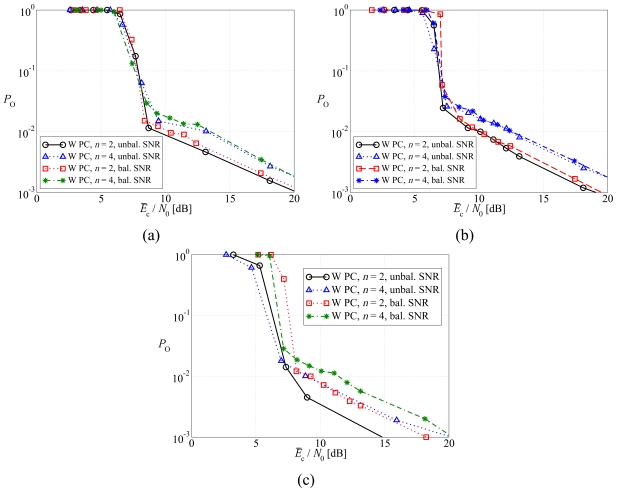
Outage probability, as a function of the average SNR at the sources, considering: (a) regular LDPC-coded schemes, (b) DD LDPC-coded schemes, and (c) SCCCed schemes. Both balanced SNR and unbalanced SNR power control strategies are considered.

In Figure 8, the outage probability is shown, as a function of the average SNR at the sources, considering (a) regular LDPC-coded schemes, (b) DD LDPC-coded schemes, and (c) SCCCed schemes. Both balanced SNR and unbalanced SNR power control strategies are considered. In this case as well, SCCC-based schemes exploit better the gain brought by the use of unbalanced SNR power control with respect to the schemes with balanced SNR power control. However, when an LDPC-based scheme is considered, this gain drastically reduces and it may happen that the unbalanced SNR power control strategy leads to a slightly worse performance.

## 5. On the Robustness of the Proposed Feedback Power Control Strategies

In this section, we investigate the robustness of the proposed feedback power control strategies against possible non-idealities. In particular, we analyze the performance in the presence of (i) mutual interference between the transmitting nodes (i.e., when the multiple access links are not perfectly orthogonal) and (ii) noisy feedback channels. Without loss of generality, a simple illustrative scenario with *n* = 2 correlated sources is considered, using either DD LDPC coding or SCCCing. However, the obtained conclusions hold also for scenarios with more than two sources.

### 5.1. Non-Orthogonal Links: Multiple Access Interference

In order to investigate the impact of non-orthogonality between the communication links, we consider the presence of mutual interference between the transmitted signals. Since an accurate characterization of the multiple access interference is beyond the scope of this paper, the residual interference is simply modeled as AWGN. Under this assumption, referring to equation (3), the real observable at the AP after matched filtering and carrier-phase estimation can be expressed as

(27) $$r_{i}^{\left(k\right)}=\left|\alpha^{\left(k\right)}\right|\sqrt{E_{\text{c}}^{\left(k\right)}}y_{i}^{\left(k\right)}+\eta_{i}^{\left(k\right)}+z_{i}^{\left(k\right)}\;i=0,\ldots ,N-1\;k=1,\ldots ,n$$

where $z_{i}^{\left(k\right)}$ is a Gaussian random variable with zero mean and variance *I_k_*/2, where

(28) $$I_{k}≜ɛ\times \sum_{j\neq k}{\left|\alpha^{\left(j\right)}\right|}^{2}E_{\text{c}}^{\left(j\right)}$$

and *ε* ∈ [0, 1] is a proper interference rejection factor. Note that the Gaussian model for the mutual interference applies accurately to non-orthogonal CDMA multiple access schemes, where *ε* is the inverse of the spreading gain [35] (e.g., see note 6). In general, we use *ε* to quantify the level of mutual interference: the higher *ε*, the higher the multiple access interference, i.e., the less orthogonal the links. In the following, the definition of average SNR at the sources will remain that given by (19), i.e., it will not take into account the interference. Therefore, for very large values of the average SNR, the only surviving noise contribution will be due to the residual interference.

**Figure 9.**
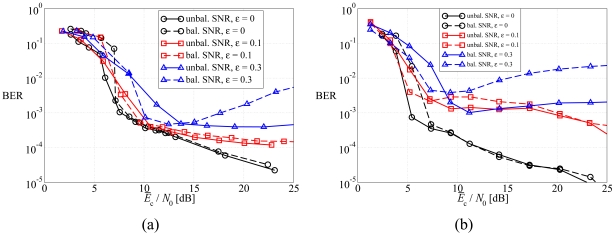
Average BER, as a function of the average SNR at the sources, in a scenario with non-orthogonal links, considering: (a) DD LDPC-coded schemes and (b) SCCCed schemes. Two possible values for *ε* are considered: (i) 0.1 and (ii) 0.3.

In Figure 9, the average BER is shown, as a function of the average SNR at the sources, in a scenario with non-orthogonal links, considering: (a) DD LDPC-coded schemes and (b) SCCCed schemes. Two possible values for *ε* are used: (i) 0.1 (moderate interference) and (ii) 0.3 (strong interference). As a reference, the curves relative to the case with orthogonal links (*ε* = 0) are also shown. As expected, the higher is *ε*, the worse is the performance, since data transmissions are affected by a larger amount of residual interference. Moreover, the unbalanced SNR power control strategy allows to obtain a performance better than that associated with the balanced SNR power control strategy for all considered values of *ε*. This effect is clearly visible in the BER waterfall region for the LDPC code, whereas it is evident at all SNRs with the SCCC, in agreement with the observation that this code is more effective in JCD schemes [25]. However, a relevant difference, with respect to the ideal case, can be observed in the presence of strong multiple access interference, namely with *ε* = 0.3. In fact, in this case the performance with balanced SNR power control worsens for increasing values of the average SNR. This is due to the fact that for large SNR the noise is basically due only to the multiple access interference. In this scenario, trying to equalize the SNRs in the two links might reverse the levels of mutual interference (from the first source to the second and vice-versa), without eliminating it. On the other hand, in the presence of unbalanced SNR power control this performance degradation, for increasing SNR, is very limited. This means that the unbalanced SNR power control strategy is more robust against multiple access interference. Finally, for *ε* = 0.3 the performance with the LDPC code is better than that with the SCCC. This suggests that channel code optimization, in the presence of multiple access interference, remains an open problem.

**Figure 10.**
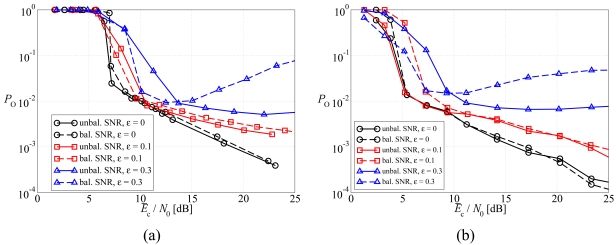
Outage probability, as a function of the average SNR at the sources, in a scenario with non-orthogonal links, considering: (a) DD LDPC-coded schemes and (b) SCCCed schemes. Two possible values for *ε* are considered: (i) 0.1 and (ii) 0.3.

In Figure 10, the outage probability is shown, as a function of the average SNR at the sources, in a scenario with non-orthogonal links, considering: (a) DD LDPC-coded schemes and (b) SCCCed schemes. As before, two possible values for *ε* are used: (i) 0.1 (moderate interference) and (ii) 0.3 (strong interference). The same considerations carried out considering the average BER still hold and, therefore, the proposed optimized unbalanced SNR power control strategy is effective for all considered values of *ε*. Moreover, the choice of the channel code is important to guarantee a desirable performance level in high interference scenarios.

### 5.2. Noisy Feedback Channels

Referring to the feedback power control commands (given by binary sequences) described in Section 3., we assume that each bit of the “up/down” binary sequence can be “flipped” with probability *P*_e−fb_. In other words, the noisy feedback links are modeled as binary symmetric channels (BSCs). Therefore, the source nodes receive power control commands which may differ from those sent by the AP. For instance, suppose that a +5 dB power control command, coded as the binary sequence “+1+1+1+1+1,” is transmitted by the AP to a source and two bits are flipped by the feedback channel, so that the command received by the source is “+1-1+1+1-1.” In this case, the received power control command is interpreted (simply by following the up/down commands) as +1 dB.

**Figure 11.**
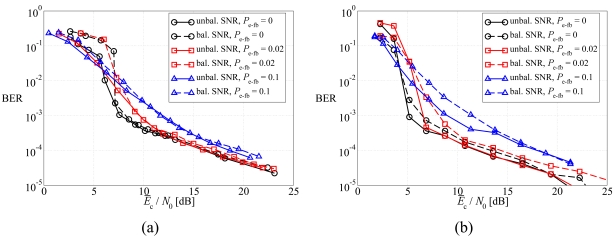
Average BER, as a function of the average SNR at the sources, in a scenario with noisy feedback channels, considering: (a) DD LDPC-coded schemes and (b) SCCCed schemes. Two possible values for *P*_e−fb_ are considered: (i) 0.02 and (ii) 0.1.

In Figure 11, the average BER is shown, as a function of the average SNR at the sources, in a scenario with noisy feedback channels, considering: (a) DD LDPC-coded schemes and (b) SCCCed schemes. Two possible values for *P*_e−fb_ are considered: (i) 0.02 and (ii) 0.1. First, one can observe that for all values of *P*_e−fb_ the unbalanced SNR power control strategy allows to obtain better performance than the balanced SNR power control strategy. In particular, in the waterfall BER region the unbalanced SNR power control strategy guarantees a noticeable performance gain with respect to the balanced SNR power control strategy. This gain reduces for large values of the SNR and, in the LDPC-coded scenario, the curves tend to overlap. This is due to the fact that the LDPC code does not effectively exploit the source correlation in the JCD algorithm. Moreover, as expected, the higher is the probability of error in the feedback channels, the worse is the performance. In particular, this effect is more pronounced for SCCCed schemes than for LDPC-coded schemes, since the SCCC better exploits the source correlation.

**Figure 12.**
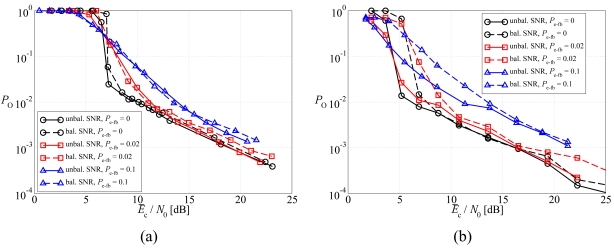
Outage probability, as a function of the average SNR at the sources, in a scenario with noisy feedback channels, considering: (a) DD LDPC-coded schemes and (b) SCCCed schemes. Two possible values for *P*_e−fb_ are considered: (i) 0.02 and (ii) 0.1.

In Figure 12, the outage probability is shown, as a function of the average SNR at the sources, in a scenario with noisy feedback channels, considering the same schemes of Figure 11. The same considerations carried out on the basis of the average BER performance still hold and, therefore, the proposed optimized unbalanced SNR power control strategy is effective for all values of *P*_fb_. Moreover, the LDPC code and the SCCC tend to behave similarly.

## 6. Extension to Scenarios with Fusion: CEO Problem

So far we have been considering general scenarios where the sources are correlated. A particular case of these scenarios can be observed when the *n* nodes correspond to sensors which observe noisy versions of the same phenomenon. The problem of detecting the single phenomenon is usually referred to as CEO problem. In particular, we assume that the same phenomenon is observed through *independent* BSCs with the same cross-over probability given by *ρ*. In this case, the correlation model between the information sequences at the input of the *n* sensors coincide with the correlation model considered for the derivation of the JCD schemes with feedback power control presented in Section 4..

**Figure 13.**
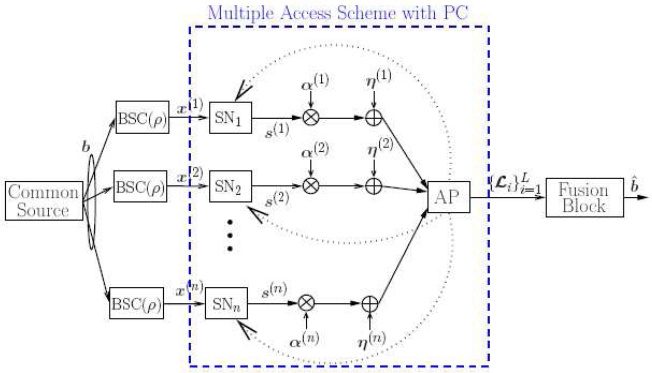
CEO scenario: multiple access scheme followed by fusion.

Under the assumption of a single common source, we consider the scheme shown in Figure 13. In this case, our goal is to use the soft-output values generated by the decoders, i.e., the LLRs at the outputs of the single component decoders, in order to estimate the sequence at the output of the common source. Note that the proposed overall scheme is given by the cascade of the multiple access scheme with feedback power control discussed in the previous sections (which remains unchanged) and a *fusion* block. In particular, both the two proposed (balanced SNR and unbalanced SNR) power control strategies can be directly applied. Therefore, comparing their performance in a CEO setting is meaningful and interesting.

In the remainder of this section, we first derive the fusion rule to be used in the corresponding block, and then we investigate the performance of the feedback power control strategies in the presence of information fusion.

### 6.1. Fusion Rule

Denote as

$${ℒ}_{i}=\left[{ℒ}_{i}^{\left(1\right)},\ldots ,{ℒ}_{i}^{\left(n\right)}\right]\;i=0,\ldots ,L-1$$

the vector of LLRs, relative to ***x****_i_*, at the output of the *n* decoders. In order to estimate *b_i_*, we consider the following maximum a posteriori (MAP) fusion rule:

(29) $${\hat{b}}_{i}≜\underset{b_{i}=0,1}{\text{arg max}\;}P(b_{i}|{ℒ}_{i})$$

The MAP strategy (29) can be rewritten, by using the total probability theorem, as

(30) $${\hat{b}}_{i}=\underset{b_{i}=0,1}{\text{arg max}\;}\sum_{\left\{x_{i}\right\}}P(b_{i}|{ℒ}_{i},x_{i})P(x_{i}|{ℒ}_{i})$$

where $x_{i}=[x_{i}^{\left(1\right)},\ldots ,x_{i}^{\left(n\right)}]$ are the noisy binary observations, relative to the *i*-th information symbol *b_i_*, at the inputs of the sensors and the sum in (30) is carried out over all possible 2*^L^* configurations for ***x**_i_*. Using the definition of conditional probability and the chain rule, the first probability at the right-hand side of (30) can be written as

(31) $$P(b_{i}|{ℒ}_{i},x_{i})=\frac{P(b_{i},{ℒ}_{i},x_{i})}{P({ℒ}_{i},x_{i})}=\frac{P({ℒ}_{i}|x_{i},b_{i})P(x_{i}|b_{i})P(b_{i})}{P({ℒ}_{i}|x_{i})P(x_{i})}$$

Since **ℒ***_i_* depend only on ***x****_i_*, owing to the considered JCD scheme, it follows that

$$P({ℒ}_{i}|x_{i},b_{i})=P({ℒ}_{i}|x_{i})$$

Moreover, since *P*(*b_i_* = 0) = *P*(*b_i_* = 1) = 1/2, from (31) one obtains

$$P(b_{i}|{ℒ}_{i},x_{i})=\frac{P(x_{i}|b_{i})P(b_{i})}{P(x_{i})}=\frac{P(x_{i}|b_{i})}{\sum_{b^{*}=0,1}P(x_{i}|b^{*})}$$

At this point, we assume that the second probability at the right-hand side of (30) can be expressed as

(32) $$P(x_{i}|{ℒ}_{i})=\prod_{j=1}^{n}P(x_{i}^{\left(j\right)}|{ℒ}_{i}^{\left(j\right)})$$

Equation (32) corresponds to assuming that the a posteriori probability of the *i*-th symbol at the input of the *j*-th source, i.e., $x_{i}^{\left(j\right)}$, depends only on the LLR, at the same epoch, generated by the corresponding decoder, i.e., ${ℒ}_{i}^{\left(j\right)}$. In other words, we assume that the a posteriori probability of $x_{i}^{\left(j\right)}$ does not depend on the other LLRs. This is reasonable, since the proposed JCD scheme exploits the existing correlation between the sources. Therefore, the LLR ${ℒ}_{i}^{\left(j\right)}$ already “embeds” the contribution from the other decoders. We remark that, in the presence of a different (e.g., non-iterative) JCD scheme, this assumption might have to be reconsidered.

Finally, the fusion rule (30) becomes

(33) $${\hat{b}}_{i}=\underset{b_{i}=0,1}{\text{arg max}\;}\sum_{\left\{x_{i}\right\}}\underset{\text{from the correlation model}}{\underbrace{\frac{P(x_{i}|b_{i})}{\sum_{b^{*}=0,1}P(x_{i}|b^{*})}}}\underset{\text{from LLRs}}{\underbrace{\prod_{j=1}^{n}P(x_{i}^{\left(j\right)}|{ℒ}_{i}^{\left(j\right)})}}$$

where we have highlighted that each addendum of the outer sum can be expressed as a product of two terms: the first one depends only on the correlation between the observations $\left\{x_{i}^{\left(j\right)}\right\}$, whereas the second one depends only on the LLRs, i.e., on the iterative JCD scheme. The probability of decision error on a single bit can then be written as

(34) $$P_{\text{e}}=\frac{1}{2}\left[P({\hat{b}}_{i}=0|b_{i}=1)+P({\hat{b}}_{i}=|1b_{i}=0)\right]$$

The evaluation of the *average* probability of decision error can be carried out through simulations, by averaging out over all transmitted packets.

It is of interest to evaluate the probability of decision error when the channel SNR becomes very high. In this case, in fact, the iterative JCD scheme allows to recover perfectly the effectively transmitted sequence, denoted as $x_{i}^{\text{corr}}$. Therefore, it follows that:

$$\underset{{\bar{E}}_{\text{c}}/N_{0}\to \infty }{\text{lim}}\;\left[\prod_{j=1}^{n}P(x_{i}^{\left(j\right)}|{ℒ}_{i}^{\left(j\right)})\right]=\{\begin{array}{cc} 1 & \text{if}\;x_{i}=x_{i}^{\text{corr}}\\ 0 & \text{if}\;x_{i}\neq x_{i}^{\text{corr}} \end{array}$$

and the fusion rule (33) becomes

(35) $$\begin{array}{cc} {\hat{b}}_{i} & =\underset{b_{i}=0,1}{\text{arg max}\;}\frac{P(x_{i}^{\text{corr}}|b_{i})}{\sum_{b^{*}=0,1}P(x_{i}^{\text{corr}}|b^{*})}\\ & =\underset{b_{i}=0,1}{\text{arg max}\;}P(x_{i}^{\text{corr}}|b_{i}) \end{array}$$

Denoting $n_{\text{b}}=n_{\text{b}}(x_{i}^{\text{corr}})$ as the number of zeros in $x_{i}^{\text{corr}}$, the probability $P(x_{i}^{\text{corr}}|b_{i})$ in (35) can be written, according to the correlation model presented in (2.1.), as follows:

(36) $$P(x_{i}^{\text{corr}}|b_{i})=\{\begin{array}{cc} {\left(1-\rho \right)}^{n_{\text{b}}}\rho^{n-n_{\text{b}}} & \text{if }b_{i}=0\\ {\left(1-\rho \right)}^{n-n_{\text{b}}}\rho^{n_{\text{b}}} & \text{if }b_{i}=1 \end{array}$$

By using (36), the decision strategy (35) becomes

$$\begin{array}{ccc} P(x_{i}^{\text{corr}}|b_{i}=0) & \begin{array}{c} \overset{{\hat{b}}_{i}=0}{>}\\ \underset{{\hat{b}}_{i}=1}{<} \end{array} & P(x_{i}^{\text{corr}}|b_{i}=1)\\ {\left(1-\rho \right)}^{n_{\text{b}}}\rho^{n-n_{\text{b}}} & \begin{array}{c} \overset{{\hat{b}}_{i}=0}{>}\\ \underset{{\hat{b}}_{i}=1}{<} \end{array} & {\left(1-\rho \right)}^{n-n_{\text{b}}}\rho^{n_{\text{b}}}\\ {\left(\frac{\rho }{1-\rho }\right)}^{n-2n_{\text{b}}} & \begin{array}{c} \overset{{\hat{b}}_{i}=0}{>}\\ \underset{{\hat{b}}_{i}=1}{<} \end{array} & 1 \end{array}$$

from which one finally obtains:

$$n_{\text{b}}\begin{array}{c} \overset{{\hat{b}}_{i}=1}{>}\\ \underset{{\hat{b}}_{i}=0}{<} \end{array}\left\lfloor \frac{n}{2}\right\rfloor$$

Note that if *n*_b_ = [*n*/2] (this can happen only if *n* is even), the decision has to be randomly taken. In this case, we arbitrarily assume that *b̂_i_* = 1. Observing that

$$\begin{array}{l} P(n_{\text{b}}=k|b_{i}=0)\;=\;\left(\begin{array}{c} n\\ k \end{array}\right)\;\rho^{n-k}{\left(1-\rho \right)}^{k}\\ P(n_{\text{b}}=k|b_{i}=1)\;=\;\left(\begin{array}{c} n\\ k \end{array}\right){\left(1-\rho \right)}^{n-k}\rho^{k} \end{array}$$

the following limits hold:

$$\begin{array}{cc} \underset{{\bar{E}}_{\text{c}}/N_{0\to \infty }}{\lim}P({\hat{b}}_{i}=0|b_{i}=1) & =P\;\left(n_{\text{b}}<\left\lfloor \frac{n}{2}\right\rfloor |b_{i}=1\right)\\ & =\sum_{k=0}^{\left\lfloor \frac{n}{2}\right\rfloor -1}\left(\begin{array}{c} n\\ k \end{array}\right)\;{\left(1-\rho \right)}^{n-k}\rho^{k}\\ \underset{{\bar{E}}_{\text{c}}/N_{0\to \infty }}{\lim}P({\hat{b}}_{i}=1|b_{i}=0) & =P\;\left(n_{\text{b}}\geq \left\lfloor \frac{n}{2}\right\rfloor |b_{i}=0\right)\\ & =\sum_{k=\left\lfloor \frac{n}{2}\right\rfloor }^{n}\;\left(\begin{array}{c} n\\ k \end{array}\right)\;{\left(1-\rho \right)}^{k}\rho^{n-k} \end{array}$$

Finally, the limiting value (for large channel SNR) of the probability of decision error (34) becomes

(37) $$P_{\text{e},\lim}≜\underset{{\bar{E}}_{\text{c}}/N_{0\to \infty }}{\lim}P_{\text{e}}=\frac{1}{2}\;\left[\sum_{k=0}^{\left\lfloor \frac{n}{2}\right\rfloor -1}\;\left(\begin{array}{c} n\\ k \end{array}\right)\;{\left(1-\rho \right)}^{n-k}\rho^{k}\;+\sum_{k=\left\lfloor \frac{n}{2}\right\rfloor }^{n}\;\left(\begin{array}{c} n\\ k \end{array}\right)\;{\left(1-\rho \right)}^{k}\rho^{n-k}\right]$$

The limiting probability of decision error in (37) corresponds to the probability of decision error in the presence of majority fusion, as typically observed in the realm of distributed detection [36], and does not depend on the channel SNR. Therefore, the probability (37) corresponds to a floor. Moreover, the final expression, at the right-hand side of (37), shows that the limiting probability of decision error *does not* depend on the particular channel code under use. However, as it will be shown in the following subsection, the chosen channel code will affect the behavior of the probability of decision error above the limiting floor. In particular, the channel code will influence the “speed” at which the floor is reached, i.e., the channel SNR at which the probability of decision error practically converges to the floor.

### 6.2. Numerical Results

**Figure 14.**
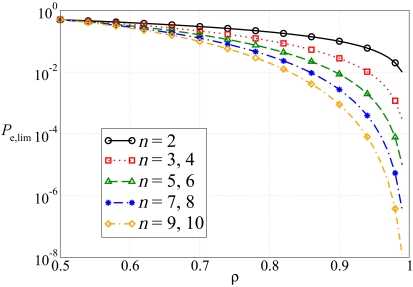
Limiting (for large SNR) probability of decision error as a function of the correlation coefficient, for the CEO scenario. Different values for the number of sensors *n* are considered.

In Figure 14, the limiting probability of decision error is shown, as a function of the correlation coefficient. Different values for the number of sensors *n* are considered. One can note that the higher is the correlation coefficient, the lower is the floor, since the correlation is better exploited and it is easier to recover the original information bit. Moreover, note that the majority decision rule does not improve when *n* increases from an odd value (e.g., 3) to the next even value (4). Therefore, no performance improvement is observed.

**Figure 15.**
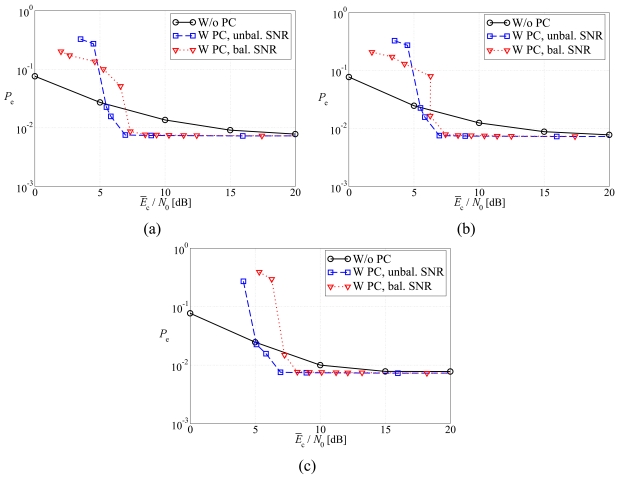
Error probability, as a function of the average SNR at the sources, for the CEO problem in a scenario with *n* = 3 sensors and considering: (a) regular LDPC-coded schemes, (b) DD LDPC-coded schemes, and (c) SCCCed schemes. The performance in the presence of balanced SNR and unbalanced SNR power control strategies is compared with that associated to the absence of power control.

In Figure 15, the probability of decision error (37) is shown, as a function of the average SNR at the sources, for the CEO problem in a scenario with *n* = 3 sensors and considering (a) regular LDPC-coded schemes, (b) DD LDPC-coded schemes, and (c) SCCCed schemes. The performance in the presence of balanced SNR and unbalanced SNR power control strategies is compared with that in the absence of power control. As anticipated from the previous subsection, all curves reach, for large values of *Ē*_c_/*N*_0_, the BER floor given by (37). Moreover, there is a difference, with respect to the results presented for non-CEO scenarios (i.e., the previously considered multiple access scheme), in the behavior of balanced SNR and unbalanced SNR power control strategies. In fact, in non-CEO scenarios the unbalanced SNR power control strategy guarantees a better performance, in terms of average BER or the outage probability, than the balanced SNR power control strategy only when an SCCC is usedm but not is an LDPC code is under use. In a CEO scenario, instead, the unbalanced SNR power control strategy allows to achieve the limiting floor faster (i.e., for lower SNRs) than the balanced SNR power control strategy in all scenarios (both SCCCed or LDPC coded).

## 7. Concluding Remarks

In this paper, we have derived feedback power control strategies and evaluated their impacts on the performance of block-faded multiple access schemes with JCD. In all cases, the use of feedback power control is expedient to set the network operational point in the feasible SNR region. First, we have derived a classical power control strategy which tries to equalize the link SNRs at the AP. Then, we have derived an innovative optimized feedback power control strategy, which allows the system operational point to lie in the feasible SNR region at the lowest overall transmit energy cost. In this case, the SNRs are typically unbalanced. Our results show that both feedback power control strategies significantly improve the performance, with respect to schemes without power control. In particular, the unbalanced SNR feedback power control strategy guarantees a performance better than that with the balanced SNR power control strategy, and this is more pronounced when a proper channel code is used (namely, a properly designed SCCC). We have then analyzed the robustness of the proposed power control strategies in the presence of non-idealities, in terms of residual multiple access interference and noisy feedback channels. Our results show that even in non-ideal scenarios the best feedback power control strategy is to unbalance the target SNRs. Finally, we have applied the proposed feedback power control strategies to a limiting case of the considered multiple access scheme, obtaining a CEO scenario where the *n* sensors make noisy observations of a common binary source. In this case, we have derived a proper fusion rule, at the AP, to be applied after power control. We have then shown that, for increasing SNR at the sensors, a limiting probability of decision error (i.e., a floor) is asymptotically reached, regardless of the channel code and feedback power control strategy under use. The feedback power control strategy, however, has an impact on the “speed,” i.e., the minimum SNR, at which this floor is reached.

## Figures and Tables

**Table 1.** Practical feedback power control commands and energy corrections. The maximum energy correction is denoted as Δ*E*_max_ (dimension: [dB]) and the minimum energy correction step is Δ*E*_step_ (dimension: [dB]).

*γ_k_* [dB]	Δ*E_c_,_k_* [dB]	Binary Feedback Command

$\left(\gamma_{k}^{\left(\text{tgt}\right)}+\Delta E_{\text{max}}-\Delta E_{\text{step}},+\infty \right]$	−Δ*E*_max_	$\underset{\Delta E_{\text{max}}/\Delta E_{\text{step}}}{\underset{︸}{-1-1\cdots -1}}$
…	…	…
$\left(\gamma_{k}^{\left(\text{tgt}\right)}+\Delta E_{\text{step}},\gamma_{k}^{\left(\text{tgt}\right)}+2\Delta E_{\text{step}}\right]$	−2Δ*E*_step_	-1-1
$\left(\gamma_{k}^{\left(\text{tgt}\right)},\gamma_{k}^{\left(\text{tgt}\right)}+\Delta E_{\text{step}}\right]$	−Δ*E*_step_	-1
$\left(\gamma_{k}^{\left(\text{tgt}\right)}-\Delta E_{\text{step}},\gamma_{k}^{\left(\text{tgt}\right)}\right]$	Δ*E*_step_	+1
$\left(\gamma_{k}^{\left(\text{tgt}\right)}-2\Delta E_{\text{step}},\gamma_{k}^{\left(\text{tgt}\right)}-1\right]$	+2Δ*E*_step_	+1+1
…	…	…
$\left(-\infty ,\gamma_{k}^{\left(\text{tgt}\right)}-\Delta E_{\text{max}}+\Delta E_{\text{step}}\right]$	+Δ*E*_max_	$\underset{\Delta E_{\text{max}}/\Delta E_{\text{step}}}{\underset{︸}{+1+1\cdots +1}}$

## Notes

1. For the sake of notational simplicity, the derivation is carried out considering a single packet transmission act, i.e., we do not use any index to indicate the specific packet.

2. Note that, for a fixed symbol duration (i.e., transmitting rate), a power variation is in a one-to-one correspondence with an energy variation.

3. Note that the internal iterations in each component subdecoder refer to (i) the iterations between the variable nodes and the check nodes in the presence of LDPC coding and the SP algorithm or (ii) the turbo iterations between convolutional decoders in the presence of turbo coding and the BCJR algorithm (at each convolutional decoder).

4. Since the a priori probabilities need to be evaluated for the systematic bits, in this case $\nu_{i}^{\left(k\right)}=x_{i}^{\left(k\right)}$ and, therefore, $\left\{P(y_{i}^{\left(1\right)},y_{i}^{\left(2\right)},\ldots ,y_{i}^{\left(n\right)})\}=\{P(2x_{i}^{\left(1\right)}-1,2x_{i}^{\left(2\right)}-1,\ldots ,2x_{i}^{\left(n\right)}-1)\right\}$. The joint PMF of ${\left\{y_{i}^{\left(k\right)}\right\}}_{k=1}^{n}$ can then be obtained directly from (1). Note that equation (24) is an approximation since, heuristically, the first probability in the summation at the right-hand side is obtained from the reliability values generated by the other decoder, whereas the second probability is a priori.

5. Note that the number of internal iterations is fixed with SCCCing, whereas it can vary in the LDPC coded case. Note also that the number $n_{\text{it}}^{\text{ext}}$ of external iterations between the component decoders differs between the cases with LDPC codes and SCCC. This is due to the different convergence characteristics of the iterative decoders associated with these channels codes.

6. Note that the exact statistics of the residual multiple access interference should be better investigated. This goes beyond the scope of this paper. However, the Gaussian approximation allows to have useful insights on the impact of the multiple access interference on the proposed feedback power control strategies.
